# Age-Related Outcomes of [^177^Lu]Lu-PSMA Radioligand Therapy in Metastatic Castration-Resistant Prostate Cancer: A Retrospective Analysis

**DOI:** 10.3390/cancers17213515

**Published:** 2025-10-31

**Authors:** Nikolaus Schweigert, Nadja Strewinsky, Daniel Köhler, Wencke Lehnert, Jonas Ekrutt, Amir Karimzadeh, Susanne Klutmann, Gunhild von Amsberg, Markus Sauer

**Affiliations:** 1 Department of Diagnostic and Interventional Radiology and Nuclear Medicine, University Medical Center Hamburg-Eppendorf, 20246 Hamburg, Germany; 2 Department of Oncology, Hematology and Bone Marrow Transplantation with Section Pneumology, Hubertus Wald-Tumorzentrum—University Cancer Center Hamburg, University Medical Center Hamburg-Eppendorf, 20246 Hamburg, Germany; 3 Martini-Klinik, Prostate Cancer Center, University Medical Center Hamburg-Eppendorf, 20251 Hamburg, Germany

**Keywords:** prostate cancer, radioligand therapy, PSMA, old, toxicity, efficacy

## Abstract

**Simple Summary:**

[^177^Lu]Lu-PSMA radioligand therapy is a well-established therapy concept for patients with metastatic castration-resistant prostate cancer. Due to multiple new early therapy regimes, there is an increasing demand, especially for older patients with secondary disease. The efficacy and toxicity of PSMA radioligand therapy are insufficiently investigated in older patients with decreased therapy tolerance, despite its general claim of a low toxicity profile. In conclusion, there is a demand for further investigations if PSMA radioligand therapy is an advisable option for patients beyond average life expectancy.

**Abstract:**

**Background/Objectives:** To investigate the efficacy and safety of treatment with [^177^Lu]Lu-PSMA-I&T Radioligand Therapy (PSMA-RLT) in older patients (≥80 years) vs. younger ones with metastatic castration-resistant prostate cancer (mCRPC). **Methods:** In this retrospective single-center analysis, 103 patients treated with PSMA-RLT between 2019 and 2024 were included. Overall survival (OS) and therapeutic response were assessed by PSA serum and based on PET/CT Imaging according to the RECIP 1.0 criteria, respectively. Toxicity was additionally assessed via laboratory (hemoglobin, cell counts, and serum creatinine). Adverse events (AEs) were detected according to CTCAE V.5. **Results:** Median OS did not differ significantly in patients ≥ 80 years vs. <80 years (13.7 vs. 16.1 months, respectively). PSA decline of ≥50% was achieved in 32% patients in total, comparably in both groups (29.4% vs. 34.8%). According to RECIP 1.0, the majority of patients with both ≥80 and <80 years demonstrated stable disease or partial responses in imaging (64% and 71%, post two cycles). Concerning toxicity, the most frequently observed AE was anemia, which occurred in both <80 and ≥80 subgroups (grade 3: 2.8% vs. 5.9%); however, no grade 4 anemia was recorded. Renal function remained stable throughout treatment, and no AE grade 3 or higher was observed. Overall, the safety profile was comparable between age groups. **Conclusions:** Treatment with PSMA-RLT can be both effective and well tolerated in patients with mCRPC aged 80 years and older.

## 1. Introduction

Prostate cancer (PCa) is one of the most frequently diagnosed cancers in men worldwide, with incidence increasing significantly with age. In 2022 alone, over 1.4 million new cases and over 396,000 deaths were reported globally [1]. While localized PCa can often be managed effectively, advanced disease, particularly metastatic castration-resistant prostate cancer (mCRPC), remains a therapeutic challenge. mCRPC is defined by disease progression despite androgen deprivation therapy (ADT), evidenced by rising prostate-specific antigen (PSA) levels, radiologic progression, and castrate serum testosterone levels [2].

Standard first-line treatments for mCRPC include androgen receptor pathway inhibitors such as abiraterone and enzalutamide. Chemotherapy with docetaxel or cabazitaxel is used in more aggressive or symptomatic cases. However, resistance to these therapies often emerges, prompting the need for alternative, targeted treatments [3,4].

Prostate-specific membrane antigen (PSMA), a transmembrane protein highly overexpressed in prostate cancer cells, has been found to be a powerful therapeutic target. Its high expression in the prostate grants the opportunity for molecular imaging (PSMA PET/CT) and targeted radioligand therapy (RLT). Among these, [^177^Lu]Lu-PSMA has shown promising results in patients with mCRPC, demonstrating favorable tumor uptake and safety in early clinical studies [5], supported by preclinical evidence of effective targeting [6]. Early real-world experience also confirmed that repeated cycles of [^177^Lu]Lu-PSMA were effective and well-tolerated [7,8].

Two landmark clinical trials have established the efficacy of [^177^Lu]Lu-PSMA. The VISION Phase III trial showed a significant improvement in progression-free and overall survival when [^177^Lu]Lu-PSMA was added to the standard of care in heavily pretreated patients [9]. Similarly, the TheraP trial demonstrated a higher PSA response rate (66% vs. 37%) and a more favorable tolerability profile with [^177^Lu]Lu-PSMA compared to cabazitaxel in a highly selective PSMA-positive/FDG-negative mCRPC cohort. However, long-term follow-up showed no significant overall survival difference between the two treatments. The patients in the collective were only selected with corresponding PSMA-positive, FDG-negative metastatic disease on dual-tracer PET imaging, resulting in a highly selective cohort [10]. The phase II PSMAfore trial demonstrated that in taxane-naïve patients with progression after androgen receptor pathway inhibitors (ARPIs), [^177^Lu]Lu-PSMA significantly improved in radiographic progression-free survival (rPFS) compared to the ARPI switch [11].

While prospective studies have consistently demonstrated the benefit of [^177^Lu]Lu-PSMA [12], evidence remains limited regarding outcomes in the oldest patients and those outside trial criteria. Recent real-world data suggest that patients not meeting the strict VISION or TheraP criteria can still benefit from [^177^Lu]Lu-PSMA therapy. A multicenter cohort study treated with [^177^Lu]Lu-PSMA reported PSA declines and survival outcomes comparable to trial-eligible patients, highlighting the relevance of studying broader populations [13].

Despite significant advances in radioligand therapy, patients aged ≥ 80 years remain underrepresented in clinical trials comparing treatment outcomes across age groups. Real-world evidence evaluating the age-stratified efficacy and safety of [^177^Lu]Lu-PSMA therapy is limited. As the proportion of elderly men with metastatic prostate cancer continues to rise, it is increasingly important to determine whether age alone should influence treatment eligibility for PSMA-RLT. In only a few previously published studies, the impact of advanced age on PSMA-RLT was analyzed; not comparing the results to the general local treatment efficacy, however, may reveal a deviation from the results of large multicenter studies like VISION.

This study aims to evaluate the efficacy and safety of [^177^Lu]Lu-PSMA in mCRPC patients stratified by age (<80 vs. ≥80 years) based on local real-world data.

## 2. Materials and Methods

### 2.1. Study Design and Population

This retrospective single-center study was conducted in accordance with the Declaration of Helsinki and approved by the local Institutional Review Board (Ethik-Komission der Ärztekammer Hamburg) with a waiver of informed consent (#1041174-WF). The analysis evaluated the efficacy and safety of [^177^Lu]Lu-PSMA-I&T radioligand therapy (PSMA-RLT) in patients with mCRPC. The study included 103 patients treated between December 2019 and September 2024. All patients received at least one cycle of [177Lu]Lu-PSMA-I&T therapy following prior failure of androgen receptor–targeted agents, taxane-based chemotherapy, or documented ineligibility for chemotherapy. No patient treated during this period was excluded from the analysis.

Eligibility was determined in an interdisciplinary tumor board and followed national guidelines for advanced prostate cancer treatment. Prior to therapy initiation, all patients underwent PSMA PET/CT imaging. PSMA expression was considered eligible for PSMA-RLT if the patient demonstrated at least one lesion of any size with PSMA uptake higher than physiological liver uptake. The indication for radioligand therapy required radiologic or biochemical progression under standard systemic therapies. Patients with severely impaired bone marrow or renal function were excluded from treatment initiation. Minimum requirements for treatment included leukocyte count > 3000/µL, thrombocyte count > 75,000/µL, serum creatinine ≤ 2 × upper limit of normal (ULN), and AST/ALT ≤ 5 × ULN. Patients were also required to have a treatment-free interval of ≥6 weeks from prior myelosuppressive chemotherapy. All patients who received at least one cycle of PSMA-RLT were systematically evaluated at baseline, ensuring the inclusion of all treated patients and minimizing selection bias.

The [177Lu]Lu-PSMA-I&T radioligand was administered intravenously every 6–8 weeks, with each cycle delivering 5.1–6.9 GBq. Treatment was continued until progression, unacceptable toxicity, or clinical deterioration. All patients remained on concurrent androgen deprivation therapy. Imaging and laboratory assessments were performed routinely to monitor efficacy and toxicity. Therapy administration and follow-up were performed according to established dosimetry and safety recommendations for [177Lu]Lu-PSMA-I&T [14], and in accordance with current international recommendations for PSMA-RLT [15].

For comparative purposes, the cohort was stratified into two age groups: patients < 80 years and patients ≥ 80 years. This stratification allowed for the evaluation of age-related differences in treatment response and tolerability, given that elderly patients are typically underrepresented in prospective clinical trials. Detailed demographic and clinical baseline characteristics are provided in Table 1.

### 2.2. Outcomes Measured

Therapeutic response was assessed using both biochemical and imaging-based criteria. PSA levels were measured following standardized recommendations (every two weeks before and in between treatment cycles). PSA response was defined as a decline of ≥50% from baseline (PSA50), with additional categories at ≥30% (PSA30) and ≥90% (PSA90). Time to progression was calculated from treatment initiation until biochemical progression, defined according to PCWG3 criteria as a PSA increase of ≥25% from the nadir and exceeding 2 ng/mL, confirmed by two sequential measurements at least three weeks apart [16]. Radiological response was evaluated using the RECIP 1.0 (Response Evaluation Criteria in PSMA Imaging in Prostate Cancer), based on PSMA PET/CT scans performed after every second therapy cycle [17]. Patients were categorized as complete response (CR), partial response (PR), stable disease (SD), or progressive disease (PD), depending on tracer uptake dynamics and tumor burden. RECIP evaluations were available for 74 patients after two cycles, 36 after four cycles, 12 after six cycles, and 1 patient after eight cycles. Safety and toxicity were monitored via laboratory assessments before each therapy cycle, including hemoglobin, platelet count, leukocytes, creatinine, alkaline phosphatase (AP), and lactate dehydrogenase (LDH). Adverse events were graded according to CTCAE version 5.0. The primary outcome was PSA50 response rate. Secondary outcomes included overall survival (OS), RECIP-based imaging response, and treatment-related toxicities.

### 2.3. Data Analysis

Descriptive statistics were used to summarize demographic, clinical, and treatment-related variables. Continuous variables, including PSA values, age, and laboratory parameters, were presented as medians and ranges. Categorical data, such as RECIP 1.0 response categories and prior treatment history, were reported as absolute numbers and percentages. PSA response rates (≥30%, ≥50%, and ≥90%) and RECIP imaging responses (CR, PR, SD, and PD) were analyzed descriptively across age groups (<80 vs. ≥80 years). Statistical significance of deviations was analyzed using chi-square tests. Disease progression rates were also assessed between the groups. Overall survival (OS) was estimated using the Kaplan–Meier method, and differences between age groups were evaluated using the log-rank test. No multivariate or regression analysis was performed. All statistical analyses were conducted using R software (R version 4.4.2 (31 October 2024)) and Microsoft Excel (Version 16.95.3 (250329)). A two-sided *p*-value of < 0.05 was considered statistically significant.

## 3. Results

### 3.1. Patient Characteristics

A total of 103 patients with metastatic castration-resistant prostate cancer were included in the analysis, with a median age of 75 years (range: 54–88); 34 patients (33%) were aged ≥ 80 years. The majority had a good performance status (ECOG 0–1 in 90.3%) and metastatic disease, involving bone (94.2%), lymph nodes (82.5%), or both. Baseline PSA levels, Gleason scores, and prior treatment exposure, including androgen deprivation therapy and novel hormonal agents, were broadly comparable between age groups. A detailed overview of clinical and demographic characteristics is provided in Table 1.

### 3.2. Prior Therapies

All 103 patients included in the study had received prior systemic treatments for mCRPC before initiation of PSMA-RLT. ADT was administered in all of the cases. A total of 56.3% of patients had previously been treated with abiraterone acetate (ABI), and 38.8% had received enzalutamide (ENZ), with 24.3% undergoing sequential therapy with both agents. A relevant amount of the study group did not receive previous treatment with chemotherapy (“off-label use” of PSMA-RLT according to local guidelines), which had been administered in 71.8% of all patients. Specifically, 70.9% had received docetaxel and 20.4% had received both docetaxel and cabazitaxel. Additional treatments included external beam radiotherapy in 29.1% of cases and radium-223 therapy in 2.9%. The majority of patients (69.9%) had received more than three prior lines of systemic therapy. When stratified by age, patients < 80 years demonstrated a higher prevalence of previous docetaxel treatment (78.3% vs. 55.9%) and combination chemotherapy (18.8% vs. 20.6%), whereas use of novel hormonal agents was more frequent among those ≥ 80 years (ABI: 64.7% vs. 52.2%; ENZ: 52.9% vs. 31.9%). Older patients were less likely to have received external beam radiation (17.7% vs. 34.8%) or combination systemic regimens. A summary of prior therapies by age group is presented in Table 1.

### 3.3. PSA Response

**Figure 1:**
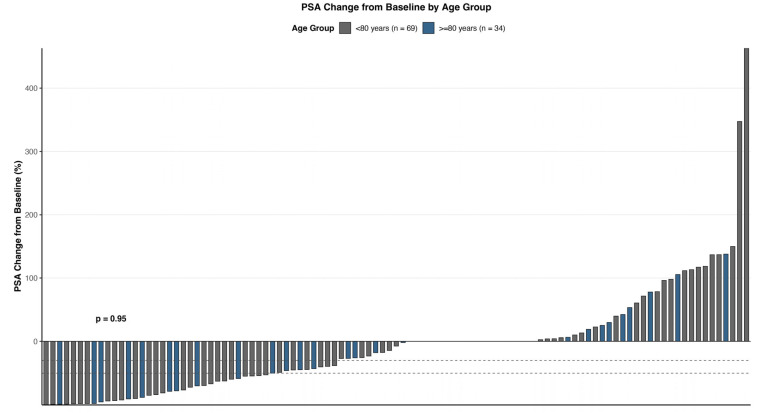
Waterfall plot of PSA change from baseline during PSMA-RLT. Gray and blue bars indicate age groups <80 years and ≥80 years, respectively.

PSA response was assessed as a biochemical marker of treatment efficacy in all 103 patients. A decline of ≥50% from baseline (PSA50) was observed in 33 patients (32.0%), while 43 patients (41.7%) achieved a reduction of ≥30% (PSA30). A higher response, defined as a ≥90% decline (PSA90), was seen in 13 patients (12.6%). A total of 26 patients (25.2%) demonstrated PSA50 within the first 12 weeks of treatment, indicating an early therapeutic effect. When stratified by age, 24 of 69 patients < 80 years old (34.8%) achieved PSA50, compared to 10 of 34 patients ≥ 80 years old (29.4%). The detected differences in response did not differ significantly (*p* = 0.64, CI: −15.7% to +26.5%). PSA30 was observed in 30 (43.5%) younger and 13 (38.2%) older patients, respectively (*p* = 0.65, CI: −18.0% to +27.5%). Among patients ≥ 80 years, 9 (26.5%) achieved PSA50 within the first 12 weeks, suggesting comparable early response rates between groups. Although PSA90 rates appeared numerically higher in younger patients (13.0% vs. 11.8%; *p* = 0.87, CI: −13.2% to +15.7%), the small sample size limited statistical comparisons. Additional analysis with respect to the impact of previous treatments with chemotherapy demonstrated comparable outcomes compared to the overall cohort: PSA50 was reached by 21.4% of younger patients vs. 33.3% in older patients without chemotherapy, respectively (*p* = 0.68). In post-chemotherapy patients, the younger patients achieved PSA50 more frequently with 38.2% vs. 26.3%; however, neither difference was significantly different with respect to statistical testing (*p* = 0.41). The median PSA at progression was notably higher in the older group (2556 ng/mL) compared to younger patients (818.98 ng/mL), although this difference likely reflects baseline PSA variability and individual disease kinetics. The average time to PSA-defined progression was longer in the ≥80 group (37.9 weeks vs. 28.8 weeks) but was based on fewer events (*n* = 4), thus limiting generalizability. The best PSA responses are visualized in Figure 1.

### 3.4. Survival Outcomes

**Figure 2:**
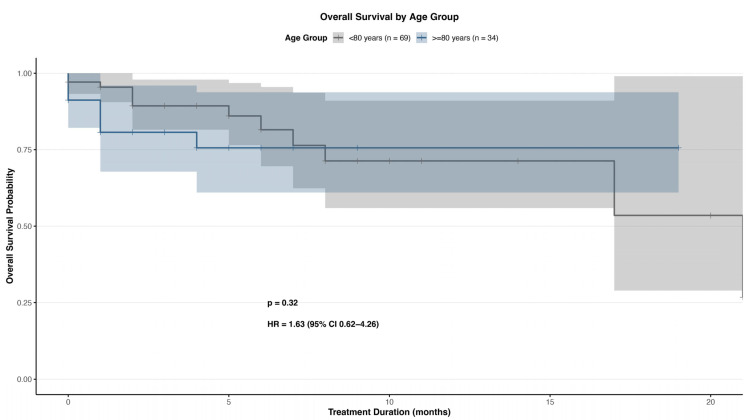
Overall survival probability. Age groups are presented in gray (<80 years) and blue (≥80 years). HR = Hazard ratio.

Overall survival (OS) was assessed in all 103 patients from the initiation of PSMA-RLT. At the time of analysis, 12 of 69 patients (17.4%) in the <80 years group and 7 of 34 patients (20.6%) in the ≥80 years group had died. The median OS for the total cohort was 15.2 months. When stratified by age, the median OS was 16.1 months in patients < 80 years and 13.7 months in patients ≥ 80 years. Kaplan–Meier analysis revealed no statistically significant difference in OS between the two age groups (log-rank *p* = 0.32). The 6-month survival probability was similar across both cohorts, and survival curves remained closely aligned throughout the follow-up period (Figure 2). These results suggest that PSMA-RLT offers a comparable survival benefit in elderly patients, including those aged ≥ 80 years. Due to the retrospective design and the relatively limited number of observed deaths, longer follow-up will be necessary to assess long-term outcomes and validate these age-stratified findings.

### 3.5. RECIP Imaging Analysis

Molecular imaging response was evaluated using [^68^Ga]Ga-PSMA-PET/CT in accordance with RECIP 1.0 criteria at regular intervals following every two cycles of PSMA-RLT, and each scan was compared to the most recent molecular imaging. A total of 74 patients underwent evaluation after two cycles, 36 after four cycles, 12 after six cycles, and 1 patient after eight cycles. Across all time points, no complete responses (CRs) were observed.

Following two treatment cycles, partial response (PR) was documented in 20 patients (27.0%), stable disease (SD) in 29 (39.2%), and progressive disease (PD) in 25 (33.8%). In patients aged ≤ 80 years (*n* = 50), PR was seen in 14 (28.0%), SD in 18 (36.0%), and PD in 18 (36.0%). Among those ≥80 years (*n* = 24), PR occurred in 6 patients (25.0%), SD in 11 (45.8%), and PD in 7 (29.2%). After four cycles, the number of evaluable patients decreased to 36. In the younger cohort (*n* = 25), PR was observed in 8 patients (32.0%), SD in 3 (12.0%), and PD in 14 (56.0%). Among the older group (*n* = 11), PR was seen in five patients (45.5%), SD in two (18.2%), and PD in four (36.4%). At the six-cycle mark, 12 patients remained evaluable, including 10 aged <80 and two ≥80. In the younger subgroup, six patients (60.0%) demonstrated PR, two (20.0%) SD, and two (20.0%) PD. Both elderly patients at this stage showed a sustained PR. Only one patient aged ≥80 received eight cycles of therapy. Imaging at this time point revealed a continued partial response, with no evidence of radiologic progression.

**Figure 3:**
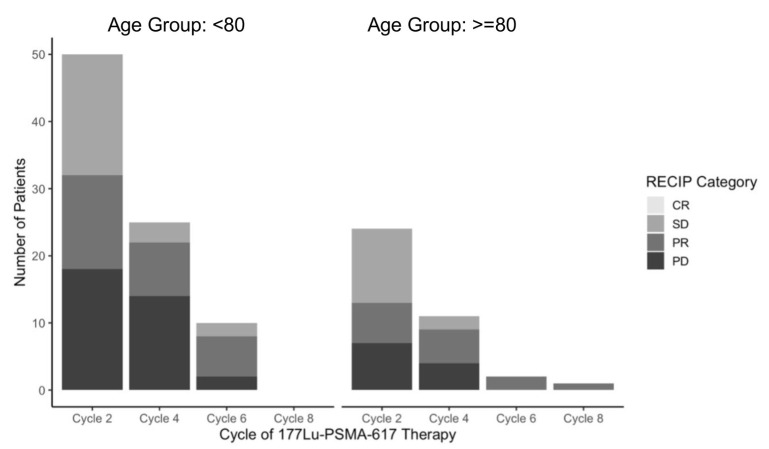
Treatment response analysis according to RECIP 1.0. CR = Complete remission. SD = Stable disease. PR = Partial remission. PD = Progressive disease.

Across all cycles, disease progression was defined according to RECIP 1.0 criteria as the appearance of new PSMA-avid lesions or a marked increase in uptake within preexisting lesions. While the number of evaluable patients declined over time, the distribution of response categories remained broadly comparable across age groups. Notably, patients ≥ 80 demonstrated stable or partial responses in the majority of cases at early cycles, and those who remained on treatment beyond four cycles often maintained disease control. All treatment responses according to RECIP 1.0 are visualized in Figure 3.

### 3.6. Toxicity Profile

Toxicity was evaluated based on routinely collected laboratory parameters and graded according to the Common Terminology Criteria for Adverse Events (CTCAE), version 5.0. The most frequently observed adverse event was anemia, which occurred in both age groups. Among patients under 80 years, 19 developed grade 1 or 2 anemia, and 2 experienced grade 3 events (2.8%), while in the group aged 80 years and older, 7 had grade 1 or 2 anemia and 2 experienced grade 3 toxicity (5.9%). No grade 4 anemia was recorded. Thrombocytopenia was observed in seven patients < 80 years and three patients ≥ 80 years as grade 1 or 2 events, and one patient in each group developed grade 3 thrombocytopenia. Neutropenia was less frequent overall, occurring in four patients < 80 years and one patient ≥ 80 years as grade 1 or 2 toxicity; one additional patient in the younger group experienced a grade 3 neutropenia. No grade 4 hematologic toxicities or febrile neutropenia were observed in either age group.

Renal function remained stable throughout treatment in both subgroups, and no patient developed a creatinine elevation corresponding to CTCAE grade 3 or higher. Alkaline phosphatase and lactate dehydrogenase levels were followed during therapy and showed variable elevations, more pronounced in patients under 80 years, though these were not further evaluated for a causal association with therapy.

**Figure 4:**
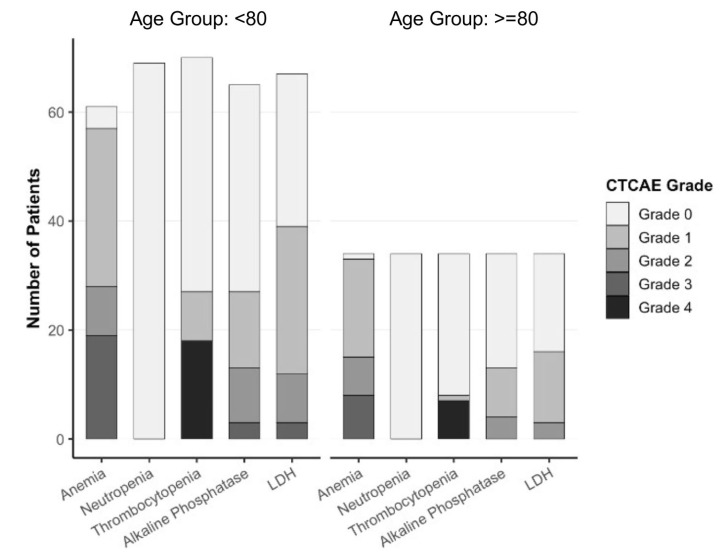
Toxicity profile analysis according to CTCAE V.5 (Common Terminology Criteria for Adverse Events).

Overall, the safety profile was comparable between age groups, with no evidence of increased toxicity among patients aged 80 years or older. No treatment-related hospitalizations or therapy discontinuations were reported during the study period. A summary of CTCAE grades by age group is provided in Figure 4.

## 4. Discussion

This retrospective single-center study evaluated the efficacy and safety of PSMA-RLT in a real-world cohort of patients with mCRPC, stratified by age above and below 80 years. Current oncology reviews emphasize the need to individualize systemic therapy in mCRPC, integrating novel radioligand approaches with established ARPIs and taxanes [18,19,20]. Given the increasing prevalence of mCRPC in elderly populations and their underrepresentation in prospective trials, this study addresses a critical gap in clinical evidence.

Biochemical response, defined as a ≥50% PSA decline, was achieved in 34.8% of patients < 80 years and 29.4% of those ≥80 years, indicating comparable PSA responses across age groups. Imaging-based outcomes, evaluated using RECIP 1.0 criteria, supported these findings: partial responses (PR) were observed in 25% of elderly patients at cycle 2, rising to 45% at cycle 4, and 100% in the small subset evaluated at cycle 6. No complete responses were observed, but a substantial proportion of patients in both age groups maintained SD or PR beyond four cycles, suggesting potential for sustained clinical benefit. While progressive disease rates increased beyond the fourth cycle, the continuation of response in selected elderly patients highlights the importance of individualized treatment beyond fixed-cycle regimens. The longitudinal RECIP assessments further emphasized therapeutic benefit, especially in elderly patients, where PSA kinetics may be less predictive, consistent with findings from Schwinger et al., who showed that combining RECIP 1.0 and PCWG3 criteria more accurately stratified overall survival in patients ≥ 80 years receiving PSMA-RLT [21]. These findings support the incorporation of molecular imaging responses into routine evaluation, particularly when biochemical markers are equivocal.

Treatment-related toxicity was generally low across age groups. Hematologic adverse events were mostly limited to CTCAE grades 1–2, and no treatment-related hospitalizations or deaths occurred. Importantly, no increase in toxicity was observed in patients ≥ 80 years, despite a higher baseline risk for treatment intolerance. This favorable safety profile reinforces the suitability of PSMA-RLT for elderly patients, including those ineligible for chemotherapy or androgen receptor-targeted agents.

Our results are consistent with the VISION and TheraP trials, which confirmed the efficacy of PSMA-RLT in mCRPC. In detail, the median overall survival of our total collective matches very closely the results of the VISION study, hence suggesting equal effectiveness of our local therapy standards. Compared to TheraP, both our and VISION’s PSA response rates underperform, which may be attributed to TheraP’s diverging inclusion criteria. A comparison of PSA response and OS is presented in detail in Table 2.

However, both VISION and TheraP underrepresented patients over 80 years, limiting generalizability to this growing patient population. In contrast, 33% of our cohort was ≥80 years, providing one of the largest elderly subgroups reported in a real-world PSMA-RLT. Deviating from both VISION and TheraP, a significant number of our patient collective was chemotherapy-naïve (28.2%), particularly within the treatment group of patients ≥80, with 43.1%. Considering that, based on local guidelines, first-line chemotherapy is, in many cases, mandatory for further treatment with PSMA-RLT, the impact of previous taxane-based therapy needs to be further addressed and is currently investigated in other trials [11]. Focusing on elderly patients ≥ 80 years in our study, we think that a relevant number of chemotherapy-naïve patients is legitimate, if not necessary, since in many cases patients would only consider best supportive care as the only acceptable alternative, and there are frequently personal reservations concerning chemotherapy. The potentially beneficial impact on the efficacy of PSMA-RLT due to a higher number of patients without previous chemotherapy in our study has to be acknowledged.

A previous study by Tauber et al. demonstrated that PSMA-RLT is both effective and well tolerated in patients ≥ 80 years [23]; however, even 61.3% of their patients were chemotherapy-naïve, which is more than in our cohort. Comparable to our findings in this study, the rate of treatment-relevant hematologic adverse events was comparable to our results, with 5% only. Considering response rates and toxicity profile, the results of Tauber et al. affirm the feasibility of PSMA-targeted therapy in older adults, comparable to our findings.

Likewise, another recently published analysis by Bastian et al. of older patients receiving PSMA-RLT demonstrated similar findings to ours, with 37.6% of patients achieving a partial response [24]. Yet the study cohort was smaller (*n* = 21) and included even older patients (≥85 years), without a reference group. Also demonstrating a similar safety profile in this study, no grade 4 adverse event was observed.

Investigating slightly younger patients (cohort ≥ 75 years), Sahin et al. demonstrated higher rates of biochemical response (61% PSA-decline of 50%), questioning higher efficacy in younger patients [25]. While no information about a local reference treatment collective was provided for this study, the toxicity profiles demonstrated comparable outcomes to our investigation.

Several limitations of our study must be acknowledged, beginning with its retrospective, single-center design, which introduces potential selection bias. Imaging follow-up intervals of all patients vary slightly due to patient noncompliance. Concerning statistical power, the size of our patient collective is limited to 103 patients, of which approx. one third was ≥ 80 years. In consequence, our group of elderly patients was smaller compared to some other studies, e.g., by Tauber et al., yet we think it is an advantage to analyze the elderly patients in the context of a control group representative for the overall treatment efficacy of our center. Median follow-up time was limited, precluding robust survival analysis, and the modest size of the ≥80 cohort limits statistical power for subgroup comparisons. Deviating from VISION and TheraP, 30% of our patients had not received prior chemotherapy, largely due to off-label use of PSMA-RLT before its formal approval in December 2022 in Germany. However, most of our results, especially OS, match the outcomes of VISION closely, and, therefore, our collective results appear to be nevertheless representative. Clinical toxicities such as fatigue, fever, or headache were not systematically captured in this analysis, and self-reported outcomes were not available. Toxicity assessment was limited to laboratory parameters graded according to CTCAE version 5.0. Furthermore, multivariate predictors of response or survival could not yet be established but may offer additional insight in future analyses.

In summary, this study adds to growing real-world evidence that PSMA-RLT may be both an effective and well tolerated in patients aged ≥ 80 years. Biochemical and imaging responses were comparable to younger patients, and no increased toxicity was observed. These findings support the inclusion of elderly patients in clinical protocols and highlight the importance of age-neutral treatment planning in mCRPC. Especially, elderly patients refusing further therapy should be provided with available information about the effectiveness and tolerance of PSMA-RLT for their respective age group by their treating physicians. Yet future prospective studies are warranted to evaluate long-term outcomes and define predictive biomarkers in this population.

## 5. Conclusions

This real-world analysis demonstrates that PSMA-RLT can be both effective and well tolerated in patients with metastatic castration-resistant prostate cancer, including those aged 80 years and older. Biochemical and imaging-based responses, as well as overall survival outcomes, were comparable between age groups, with no significant increase in treatment-related toxicity among elderly patients. These findings suggest that chronological age alone should not be considered a limiting factor in the decision to offer PSMA-targeted therapy. Given the increasing prevalence of prostate cancer in aging populations, prospective studies specifically designed to assess the efficacy, safety, and quality-of-life impact of PSMA-RLT in elderly and comorbid patient populations are important.

## Figures and Tables

**Table 1:** Patient characteristics.

	All Patients (*n* = 103)	<80 Years (*n* = 69)	≥80 Years (*n* = 34)
	Median (Range)/*n* (%)	Median (Range)/*n* (%)	Median (Range)/*n* (%)
Age	75 (54–88)	71 (54–79)	82 (80–88)
ECOG Performance Status Score of 0 or 1–Nr. (%)	93 (90.29%)	63 (91.30%)	30 (88.24%)
Sites of Metastasis—*n* (%)			
Primary Metastasis	96 (93.20%)	65 (94.20%)	31 (91.18%)
Pelvic LN	31 (30.10%)	21 (30.43%)	10 (29.41%)
Extrapelvic LN	53 (51.46%)	36 (52.17%)	17 (50.00%)
LN Only	2 (1.94%)	1 (1.45%)	1 (2.94%)
Bone	97 (94.17%)	64 (92.75%)	33 (97.06%)
Bone + LN	62 (60.19%)	38 (55.07%)	24 (70.59%)
Bone Only	32 (31.07%)	25 (36.23%)	7 (20.59%)
Visceral	12 (11.65%)	6 (8.70%)	6 (17.65%)
Lung	11 (10.68%)	6 (8.70%)	5 (14.71%)
Liver	3 (2.91%)	0 (0.00%)	3 (8.82%)
Adrenal	4 (3.88%)	3 (4.35%)	1 (2.94%)
CNS	0 (0.00%)	0 (0.00%)	0 (0.00%)
Visceral Organs Only	0 (0.00%)	0 (0.00%)	0 (0.00%)
PSA Level ng/mL (Range)	266.67 (0.02–2782.43)	201.09 (0.25–2484.05)	137.27 (0.02–2782.43)
Alkaline Phosphatase IU/Liter (Range)	178.36 (43.00–1422.00)	200.77 (44.00–1422.00)	132.88 (43.00–561.00)
LDH IU/Liter (Range)	314.17 (14.00–1974.00)	334.70 (14.00–1974.00)	272.53 (161.00–537.00)
Gleason Score—*n* (%)			
7	31 (30.10%)	23 (33.33%)	10 (29.41%)
8–10	48 (46.60%)	35 (50.72%)	13 (38.24%)
Unknown	21 (20.39%)	10 (14.49%)	11 (32.35%)
Prior Therapies—*n* (%)	398	276	122
ADT	100 (97.09%)	67 (97.10%)	33 (97.06%)
ABI	58 (56.31%)	36 (52.17%)	22 (64.71%)
ENZ	40 (38.83%)	22 (31.88%)	18 (52.94%)
ABI + ENZ	25 (24.27%)	12 (17.39%)	13 (38.24%)
DOCE	73 (70.87%)	54 (78.26%)	19 (55.88%)
CABA	21 (20.39%)	14 (20.29%)	7 (20.59%)
DOCE + CABA	21 (20.39%)	14 (20.29%)	7 (20.59%)
CTX	74 (71.84%)	55 (79.91%)	19 (55.88%)
RTX	30 (29.13%)	24 (34.78%)	6 (17.65%)
Ra223	3 (2.91%)	1 (1.45%)	2 (5.88%)
Prior Therapies > 3	72 (69.90%)	56 (84.06%)	23 (67.65%)

**Table 2:** Comparison of PSA50 Response and Median Overall Survival (OS) with the VISION Trial [9] and the TheraP Trial [10,22].

	All Patients	<80 Years	≥80 Years	VISION	TheraP
PSA50 Response	32%	35%	29%	46%	66%
Median OS (Months)	15.2	16.1	13.7	15.3	16.4

## Data Availability

The datasets used and/or analyzed during the current study are available from the corresponding author upon reasonable request.

## Abbreviations

The following abbreviations are used in this manuscript:

[177Lu]Lu-PSMA-617	Lutetium-177–Prostate-Specific Membrane Antigen 617
[177Lu]Lu-PSMA-I&T	Lutetium-177–Prostate-Specific Membrane Antigen I&T
ABI	Abiraterone acetate
ADT	Androgen deprivation therapy
ALT	Alanine aminotransferase
AP	Alkaline phosphatase
ARPI	Androgen receptor pathway inhibitor
AS	Aspartate aminotransferase
CABA	Cabazitaxel
CN	Central nervous system
CR	Complete response (per RECIP 1.0 criteria)
CTX	Chemotherapy
CTCAE	Common Terminology Criteria for Adverse Events (version 5.0)
DOCE	Docetaxel
ECOG	Eastern Cooperative Oncology Group performance status
ENZ	Enzalutamide
FD	Fluorodeoxyglucose
GBq	Gigabecquerel
LD	Lactate dehydrogenase
LN	Lymph node
mCRPC	Metastatic castration-resistant prostate cancer
OS	Overall survival
PCa	Prostate cancer
PCWG3	Prostate Cancer Clinical Trials Working Group 3
PD	Progressive disease (per RECIP 1.0 criteria)
PET/CT	Positron emission tomography/Computed tomography
PR	Partial response (per RECIP 1.0 criteria)
PSA	Prostate-specific antigen
PSA30	Reduction in PSA of ≥30% from baseline
PSA50	Reduction in PSA of ≥50% from baseline
PSA90	Reduction in PSA of ≥90% from baseline
PSMA	Prostate-specific membrane antigen
Ra223	Radium-223 dichloride
rPFS	Radiographic Progression-Free Survival
RECIP 1.0	Response Evaluation Criteria in PSMA Imaging in Prostate Cancer (version 1.0)
RLT	Radioligand therapy
RTX	Radiotherapy
SD	Stable disease (per RECIP 1.0 criteria)
ULN	Upper limit of normal
